# Authenticity of Honey: Characterization, Bioactivities and Sensorial Properties

**DOI:** 10.3390/foods11091301

**Published:** 2022-04-29

**Authors:** Olga Escuredo, M. Carmen Seijo

**Affiliations:** Department of Vegetal Biology and Soil Science, Faculty of Science, University of Vigo, As Lagoas, 32004 Ourense, Spain; mcoello@uvigo.es

Honey is a natural product well known for its beneficial properties, which depend on its composition. This composition of honey is related to its botanical and geographical origin, as well as its environmental and management conditions. Honey contains a wide spectrum of components, many of which are phytochemicals found in plants; these include compounds with highly demonstrated antimicrobial, antioxidant and anti-inflammatory properties. Studies about the composition, properties, and botanical and geographical origins of different honey types are relevant in society; they are especially relevant in the honey market, as they provide knowledge to guarantee honey authenticity. 

For years, the determination of the geographical and botanical origin of honey has been based on the microscopic examination of its pollen profile. However, due to a lack of specialists in palynology, there is a tendency to replace pollen analysis with instrumental methods of finding biochemical markers for honey discrimination. Nevertheless, studies on unifloral honey, particularly if they have similar physicochemical characteristics, need to be complemented with palynological information to successfully discriminate its botanical origin. Research based on botanical and geographical origin that delves into the chemical, physical and health qualities—as well as the pollen profile—of honey as food is needed, to valorize the product in the market both locally and internationally. 

In this Special Issue, our aim is to publish research papers on the authenticity, characterization, and biological properties of honey. Thus, various aspects of its physicochemical composition, quality parameters, sensorial profile, functional properties, healthy compounds, palynological characteristics, and the relationships of these factors with their botanical and geographical origins, are discussed. Methods to identify the entomological origin and possible adulteration of honey are also considered. The papers selected for this Special Issue were subject to a rigorous peer review procedure, with the aim of rapid and wide dissemination of research results, and their transfer to different stakeholders and the scientific community.

The paper presented by Labsvards et al. [1] assessed the chemical profiles of 78 honeys from Latvia (buckwheat, clover, heather, linden, rapeseed, willow, and polyfloral honey) using light stable-isotope-ratio mass spectrometry (IRMS), ultra-high-performance liquid chromatography coupled with high-resolution mass spectrometry (UHPLC-HRMS), and nuclear magnetic resonance (NMR) methods. The researchers validated the floral origin of honeys using melissopalynology. The study showed a combination of multiple analytical and statistical methods to differentiate between various types of monofloral honey, and to find adequate indicators for the classification of their botanical origins. Therefore, this study reports on the botanical authentication and quality of honey from this geographical origin.

The study carried out by Vázquez et al. [2] showed that the combination of chromatographic analysis with mass spectrometry detection and principal component analysis are adequate to investigate the botanical authentication of honey from Northwest Spain. A miniaturized, fast and environmentally friendly experimental procedure (VE-UAE) based on liquid chromatography–tandem mass spectrometry (LC-MS/MS) was successfully developed. Specifically, the statistical treatment—based on concentrations of total phenols, antioxidant activity and individual phenolic compounds—revealed significant differences according to the studied honey type, demonstrating that phenolic compounds can be used as indicators to identify botanical origins.

A melissopalynological analysis and some physicochemical parameters of 45 unifloral honeys (raspberry, mint, sunflower, thyme and rape) and polyfloral honeys from different regions of Romania were analyzed by Pauliuc et al. [3]. Principal component analysis confirmed the possibility of the botanical authentication of rape, sunflower and thyme honey samples based on the following physicochemical parameters: moisture content, pH, free acidity, electrical conductivity, hydroxymethylfurfural content, color, total polyphenol content, flavonoid content, DPPH radical scavenging activity, phenolic acids, flavonols, sugars and organic acids. However, the mint honey and raspberry honey were not satisfactorily separated. The researchers highlighted sugars, individual phenolic compounds and organic acids as the least influential compounds in the botanical origin of the studied honey types. It is important to note that for better classification of honey, it is necessary to study a large number of samples from different botanical origins.

Homrani et al. [4] characterized 62 honey samples produced in different bioclimatic areas of Algeria using palynology and their physicochemical properties. This paper highlighted the great diversity of honey production in Algeria, evidencing the importance of honey characterization to guarantee authenticity and to valorize local production. Some botanical taxa important for honey production, such as *Eucalyptus*, *Brassica napus*, *Hedysarum* and *Citrus*, are common honey plants in Mediterranean areas. However, others such as *Capparis spinosa*, *Asparagus*, *Ziziphus lotus* and some Apiaceae plants, are representative of the honey from this country, and are useful as markers to guarantee their authenticity. Based on the combination of palynological characteristics, physicochemical parameters and sensorial properties with statistical analysis, Ghorab et al. [5] differentiated 30 honey samples, produced in Babors Kabylia (North east of Algeria), by botanical origin. The pollen spectrum performed revealed a great diversity in pollen types (96 pollen types), with the main pollen types being the spontaneous species Fabaceae (*Hedysarum*, *Trifolium*, Genisteae plants), Asteraceae plants, Ericaceae (mainly *Erica arborea*), *Myrtus* and *Pistacia*. This is the first study to focus on sensory properties and their relationships with the botanical origin and the physicochemical properties of honey from Algeria. Considering that this area is a hotspot for biodiversity due to its high number of endemic plants, this study evidenced the wide variety of honey types that can be obtained in the area. Further studies could contribute to an increase in knowledge about these honey types and the most relevant plant species for honey yield, and could contribute to the valorization of local beekeeping.

Bucekova et al. [6] reported the need to specify some additional standards on the biological properties of honey. This study focused on evaluating the antibacterial activity, the content of hydrogen peroxide (H_2_O_2_) and the protein profile of 36 commercial honeys purchased in Slovakia, both from local beekeepers and medical-grade (recommended for treating infected wounds). More than 40% of the commercial honeys purchased presented low antibacterial activity, which is indicative of artificial honey (only sugars). In general, the honey samples exhibited high antibacterial activity, while generating low levels of H_2_O_2_. On the other hand, the honey samples from local beekeepers showed superior antibacterial activity compared to the medical-grade honeys. Tsavea et al. [7] demonstrated the ability of pine honeydew samples produced across Greece to generate high levels of H_2_O_2_, even higher than other types of honeydew honey. Furthermore, due to their high polyphenol content, a strong antioxidant activity of the honey samples was also denoted. The breakdown of H_2_O_2_, using a catalase treatment, into a honey solution resulted in a significant decrease in antibacterial activity. Similarly, the digestion of honey proteins by proteinase K resulted in lower antibacterial efficacy among the studied honey samples, depending, again, on the specific bacteria. Thus, the results suggest multiple underlying mechanisms of the antibacterial activity of pine honeydew honeys. These researchers demonstrated that the antibacterial activity of honey can be easily altered using adulteration, thermal treatment or prolonged storage; therefore, it fulfils strict criteria to be a suitable new quality standard [6,7]. 

Sun et al. [8] investigated the chemical composition and the biological and anti-inflammatory activities of Safflower honey from Northwest China, produced using nectar from the medicinal plant *Carthamus tinctorius*. The extract of this medicinal plant was studied as it has very interesting biological activity properties, but the chemical and biological composition of honey from this botanical origin has been poorly evaluated. The results of the study revealed a great capacity to capture DPPH and ABTS+ free radicals of Safflower honey in vitro [8]. The special antioxidant and anti-inflammatory properties of safflower honey revealed its potential as a novel functional food. Despite being a little-studied honey, the future of this type of honey is encouraging due to its biological qualities and excellent nutritional value.

Xagoraris et al. [9] analyzed the main volatile compounds of 25 autumn heather honeys from the indigenous Greek *Erica manipuliflora* using solid-phase microextraction (SPME) methodology, followed by gas chromatography–mass spectrometry (GC-MS). This is the first study to identify a volatile profile of honey from *E. manipuliflora*. Optimal method conditions were proposed for all the dominant volatile compounds, and predictive models were provided to evaluate each volatile compound separately. The objective of this study was to reinforce the characterization of this honey type. 

Non-destructive characterization tools for quality control and the physicochemical compounds of honey, based on the principles of spectroscopy, are considered in this Special Issue [10]. Prediction models using portable near-infrared (MicroNIR) spectroscopy and reference physicochemical parameters of honey were developed to authenticate 100 honey samples from Northwest Spain. Using multivariate and partial least square regressions, the authors developed excellent models for moisture, hydroxymethylfurfural, color and total flavonoids, as well as acceptable statistics for electrical conductivity, pH and total phenols. Nevertheless, further experiments are proposed, to build a robust database that could support the use of this equipment as a quick alternative for honey authentication. 

In addition to the identification of pollen grains, a great variety of particles in honey sediment can be found using microscopic analysis. Magyar et al. [11] identified some airborne particles present in honey from Poland and Tunisia, which provided information for the identification of its geographical origin. The study was based on the high percentage of anemophilous pollen grains and spores, and the great variety of particles found in the honey sediment. The presence of siliceous marine microfossils was related to the characteristics of the areas in which the honey was produced. The silicoflagellates are deposited from the air onto nectareous flowers and, consequently, bees transport them to their hives. These authors concluded that the silicoflagellates could be used as complementary indicators of the geographical origin of honeys collected in areas characterized by diatomite outcrops. This is the first document that provides a record of microfossils in honey, represented by silicoflagellates, diatoms and endoskeletal dinoflagellates.

Finally, this Special Issue includes a study on the importance of differentiating the entomological origin of honey [12]. *Apis mellifera* honey, *A. cerana* honey and *A. dorsata* honey are the three dominant honey types in the Asian market. Some studies indicate that *A. dorsata* honey has higher antioxidant properties and medical values than *A. mellifera* honey. *A. dorsata* honey and *A. cerana* honey are vulnerable to adulteration (as a result of false geographical and botanical origin, or from mixing their composition with sugars or syrups), due to the higher price in the market compared to *A. mellifera* honey [12]. Therefore, it is important to develop fast, reliable and cost-effective identification methods to determine the honey’s origin. Mohamadzade et al. [12] proposed a rapid and accurate PCR-based method. In this study, three species-specific primers were designed to amplify the short part of the *NADH dehydrogenase 2* (*ND2*) region of mtDNA. At the same time, with this method, the authors detected a mixture of *A. mellifera* honey with *A. dorsata* and *A. cerana* honey. Additionally, honey samples from different countries were used to evaluate the accuracy of the developed method. 

In summary, the papers published in this Special Issue cover different analytical procedures for the identification of the botanical and geographical origins of honey. Authentication of the botanical and geographical origin of honey requires multiple analytical, statistical and mathematical methods for the determination of chemical compounds, and also requires the characterization of its sensory properties and pollen profile. The papers aim to report the sensory, chemical, nutritional, health and physical qualities of honey produced in different regions worldwide. New technologies looking for different purposes were even successfully adapted to the matrix of honey. The global trend towards healthy foods with high nutritional value arouses curiosity and sparks multidimensional and multidisciplinary approaches, as shown in the documents described above. The authors who have contributed to this issue inform the scientific community about the problem of, and the interest in, the authentication of honey as a food, which will undoubtedly help to deepen future studies.
